# Book Reviews

**Published:** 1911-07

**Authors:** 


### BOOK REVIEWS

THE ANATOMIC HISTOLOGICAL PROCESSES OF BRIGHT'S DISEASE. By Horst Oertel, M.D., Director of the Russell Sage Institue of Pathology, New York. Octavo of 227 pages, with 44 illustrations and 6 lithographic plates. Philadelphia and London: W. B. Saunders Company, 1911. Cloth, $5.00 net.

The reviewer of this book always enjoys reading a monograph and especially when so many and important facts are correlated and systematized in such a manner as to give a clearer conception of such a changing and variable disease as is Bright's disease. It deals with the morphology of nephritis in a way which emphasizes relations and reconstructs the whole as a unit of interwoven processes.

We most heartily commend it.

DIAGNOSTIC AND THERAPEUTIC TECHNIC. By Albert S. Morrow, Adjunct Professor of Surgery, New York Polyclinic. Octavo of 850 pages, with 815 original line drawings. Philadelphia and London: W. B. Saunders Company, 1911. Cloth, $5.00 net.

Morrow has brought together and arranged in a manner easily accessible for reference a large number of procedures employed in diagnosis and treatment. The majority of the procedures are every-day practical methods of interest to the general practitioner.

---

VAGINAL CELIOTOMY. By S. Wyllis Bandler, M.D., Fellow of the American Association of Obstetricians and Gynecologists, Adjunct Prof. of Diseases of Women, New York Post Graduate Med. School. 148 illustrations. Published by W. B. Saunders & Co., Philadelphia.

The importance of this method of performing hysterectomy has undoubtedly been neglected and we are glad to see such an excellent presentation of the entire subject as is to be found in Bandler's monograph. The drawings, subject-matter and general make-up are well above the average.

---

MERCK'S MANUAL OF THE MATERIA MEDICA. (Fourth Edition). A Ready Reference Pocket Book for the Physician and Surgeon. Containing a comprehensive list of Chemicals and Drugs—not confined to "'Merck's"—with their synonyms, solubilities, physiological effects, therapeutic uses, doses, incompatibles, antidotes, therapeutic Indications, with interspersed paragraphs on Bedside Diagnosis, and a collection of Prescription Formulas, beginning under the indication "Abortion" and ending with "Yellow Fever;" a Classification of Medicaments; and Miscellany, comprising Poisoning and Its Treatment; and an extensive Dose Table; a Chapter on rinalysis and various tables, etc., etc. (Merck & Co., 45 Park Place, New York, 1911. 493 pages. Sent on receipt of forwarding charges of 10 cents, in stamps, to physicians, or to students enrolled in any College of Medicine, in the United States).

A TEXT-BOOK OF SURGICAL ANATOMY. By William Francis Campbell, M.D., Professor of Anatomy at the Long Island College Hospital. Second edition revised. Octavo of 675 pages, with 319 original illustrations. Philadelphia and London: W. B. Saunders Company, 1911. Cloth $5.00 net; half morocco, $6.50 net.

The author has presented in this book the essentials of practical economy and has emphasized the parts on the regions of peculiar interest to the surgeon. The success accorded the first edition of this work showed there was a distinct demand for such anatomic facts.

